# Adolescent psychiatric outpatient care rapidly switched to remote visits during the COVID-19 pandemic

**DOI:** 10.1186/s12888-021-03580-w

**Published:** 2021-11-20

**Authors:** Emma M. Savilahti, Sakari Lintula, Laura Häkkinen, Mauri Marttunen, Niklas Granö

**Affiliations:** 1 Adolescent Psychiatry, University of Helsinki and Helsinki University Hospital, PO BOX 660, 00029 HUS Helsinki, Finland; 2 Mental Health Unit, National Institute for Health and Welfare, Helsinki, Finland

**Keywords:** COVID-19, Pandemic, Adolescent psychiatry, Outpatient, Remote visit

## Abstract

**Background:**

The COVID-19-pandemic and especially the physical distancing measures drastically changed the conditions for providing outpatient care in adolescent psychiatry.

**Methods:**

We investigated the outpatient services of adolescent psychiatry in the Helsinki University Hospital (HUH) from 1/1/2015 until 12/31/2020. We retrieved data from the in-house data software on the number of visits in total and categorized as in-person or remote visits, and analysed the data on a weekly basis. We further analysed these variables grouped according to the psychiatric diagnoses coded for visits. Data on the number of patients and on referrals from other health care providers were available on a monthly basis. We investigated the data descriptively and with a time-series analysis comparing the pre-pandemic period to the period of the COVID-19 pandemic.

**Results:**

The total number of visits decreased slightly at the early stage of the COVID-19 pandemic in Spring 2020. Remote visits sharply increased starting in 3/2020 and remained at a high level compared with previous years. In-person visits decreased in Spring 2020, but gradually increased afterwards. The number of patients transiently fell in Spring 2020.

**Conclusions:**

Rapid switch to remote visits in outpatient care of adolescent psychiatry made it possible to avoid a drastic drop in the number of visits despite the physical distancing measures during the COVID-19 pandemic.

**Supplementary Information:**

The online version contains supplementary material available at 10.1186/s12888-021-03580-w.

## Background

World Health Organization (WHO) declared on 3/11/2020 that the SARS-CoV-2 virus, which causes COVID-19, had spread to a pandemic. The pandemic with the ensuing physical distancing measures has drastically impacted social life, economic circumstances and personal freedom. Adolescents are especially vulnerable to the disruptions given their dependence on their care-givers and their developmental tasks that may be severed by lack of adequate social, emotional and educational stimuli and support [1, 2]. Research on mental health during the pandemic was first published predominantly on adult populations, but data on children and adolescents have begun to emerge. Few studies have, however, reported on how adolescent psychiatric services have adapted to the unprecedented circumstances.

Numerous studies based on self-assessment surveys have reported signs of poor mental health in adult general populations during the first wave of the COVID-19 pandemic [3, 4]. In contrast, a Dutch longitudinal study observed no significant increase in depressive and anxiety symptoms in 3/2020 compared with 11/2019, but did find a slight decrease in symptoms in 6/2020 compared with both previous time points [5].

Although studies on mental health of children and adolescents during the COVID-19 pandemic are far fewer than on adult populations, they have reported nuanced observations. A German nationwide survey of 7–17-year olds showed that compared to pre-pandemic results from another nationwide cohort study, subjects had more mental health problems than before the pandemic based on both their own and their parents’ reporting [6]. Two studies from the USA observed that mental health symptoms in adolescents increased in Spring 2020 during the first wave of the pandemic and then subsided towards the Summer, when the pandemic and ensuing restrictions eased [7, 8]. In a Canadian study on both community and psychiatric clinical populations, the majority (70%) of parents reported in 4–6/2020 that the mental health of their 6–18 year old child had deteriorated with stress related to social isolation, whereas 20% of parents reported improvement in their child’s mental health [9].

Mental health services are challenged during the pandemic by physical distancing measures as well as potential changes in demand. In contrast to the reports that mental health indicators have deteriorated at the onset of the COVID-19 pandemic, studies on mental health care have observed sharp decreases in demand of services [10–12]. Psychiatric emergency visits in the early stage of the pandemic (Spring 2020) were fewer than before the pandemic according to seven studies recently reviewed [11] and in a study on children and adolescents (under 18 years of age) with data from ten countries [12]. In a French study on adults, the proportion of referrals due to psychosis and on involuntary basis increased, whereas visits due to anxiety disorders and first psychiatric contacts were lower than in 3–4/2019 [13]. In primary health care in the UK in 4/2020, the incidence of depression and anxiety disorders had reduced by nearly half, and the rate of referral to mental health services was less than a quarter compared with expected rates based on data from previous 10 years [10]. By 9/2020, however, the incidence of several mental health problems had increased to expected levels in England, while elsewhere in the UK rates remained around a third lower than expected [10]. In secondary mental health care services the overall number of registered patients decreased from 4/2020 to 9/2020 compared with pre-pandemic period, whereas the number of underaged patients slightly increased [14]. The total number of clinical contacts for underaged patients in Spring 2020 reduced less than for adults, and while in-person visits decreased, remote visits increased [14].

Our aim was to investigate how adolescent outpatient psychiatric care in Helsinki University Hospital has changed during the pandemic from its onset until the end of year 2020 compared with previous 5 years. We are aware of only one previous study reporting on adolescent outpatient psychiatric care during the pandemic [14], and no previous studies have, to our knowledge, investigated whether changes in adolescent outpatient psychiatric care differed between diagnostic groups. The extant research on mental health during the COVID-19 pandemic provides discordant basis for hypotheses: psychiatric symptoms increased, whereas visits to health care services for psychiatric reasons decreased. Firstly, we hypothesized that the overall number of outpatient visits in adolescent psychiatry dropped during the first lockdown in Spring 2020, consistent with the changes reported in the UK primary health care [10] and in psychiatric emergency visits in several countries [12, 13]. Secondly, we expected visits to increase after Spring 2020 to attain the previous or possibly an even higher level, considering that in specialized adolescent psychiatric services most of the patients suffer from mid- to long-term problems and that other studies indicate that mental health has deteriorated in both adult [3, 4] and underage [6–9] populations during the pandemic. Thirdly we further expected an increase in remote visits and a decrease in in-person visits during the pandemic especially concomitantly with the lockdown in Spring 2020. Finally, we hypothesized most changes in the number of visits to be in the diagnostic group of depressive and anxiety disorders and least in psychotic disorders due to the severity of these disorders, and based on observations from a French study [13].

## Methods

### Setting

In Finland, the spread of the virus started later than in many other European countries. Finnish health officials recommended physical distancing measures starting in 3/2020. The first lockdown started on 3/16/2020. Primary and secondary schools were operating remotely 3/17–5/13/2020 except for some pupils with special needs. High schools and vocational schools operated remotely from 3/17/2020 until the end of the semester (beginning of June). The second wave in Autumn/Winter 2020–2021 resulted in less severe restrictions. Primary and secondary schools did not move to remote learning, but high schools and vocational schools operated remotely from 11/30/2020 to Spring 2021.

The department of adolescent psychiatry of the Helsinki University Hospital (HUH) is responsible for the publicly funded specialized psychiatric services for 13–17-year old residents (*n* = 92,677 in 2020) of the Uusimaa district (population 1.71 million in 2021) in Southern Finland. Due to the physical distancing measures nationally installed to mitigate the spread of COVID-19 in Finland, HUH adolescent psychiatry moved to supplying outpatient services predominantly remotely from 3/17/2020 onwards. After the lockdown from 3/16/2020 to 5/13/2020, remote services were still recommended except in emergency situations, but patients had a subjective right to choose in-person visits. Wearing of masks at in-person visits became mandatory in HUH clinics from 9/1/2020 onwards.

The study was approved by the research administrative board of the department of psychiatry at HUH (decision number HUS/153/2021), and conducted at the division of adolescent psychiatry, HUH, Finland. Since we did not gather or analyse any identifiable patient data, review by an ethical board was not necessary.

### Data

We investigated outpatient visits and referrals in the division of adolescent psychiatry of HUH from 1/1/2015 until 12/31/2020. We chose to retrieve the data starting on 1/1/2015 in order to have a relatively long reference period preceding the pandemic. We retrieved data from the in-house hospital data software (called HUS Total) on a daily basis on the number of visits in total as well as separately categorized as in-person visit or remote visit (phone calls and video calls over internet) and on a monthly basis on the number of persons in outpatient care.

We further analysed these variables according to the psychiatric diagnoses coded for each visit. Psychiatric diagnoses were coded in the system by clinicians according to ICD-10. For analyses, we combined diagnoses into the following broad four groups: 1) psychotic disorders (F20-F29), 2) depressive and anxiety disorders (“neurotic” F32, F33, F40–48, F93.0, F93.1, F93.2, F93.80, F93.9), 3) ADHD and conduct disorders (F90, F91, F92), and 4) all other psychiatric diagnoses not included in the previous categories. Primary, severe eating disorders in HUH are treated in a specialized unit affiliated to adult psychiatry, and are thus not in the data on adolescent psychiatric outpatient care. Eating disorders in our data are included in the category of other diagnoses. We aggregated the daily data to weekly time series data.

Data on referrals to adolescent psychiatric out-patient care from other health care providers (mostly from primary health care) were available on a monthly basis. The data on referrals did not include referrals or visits to emergency services.

Based on the public statements and regulations of the Finnish government and health care authorities, we defined the start of the COVID-19 pandemic period with widely implemented physical distancing measures at 3/16/2020 and the lockdown period 3/16/2020–5/18/2020.

### Data analyses

We investigated the data descriptively and with regression models and time-series analyses with emphasis on comparing the pre-pandemic period (in Finland 1/1/2015–3/15/2020) to the period of the COVID-19 pandemic (from 3/16/2020 until the end of study period 12/31/2020).

The regression analysis of our count data was done using quasi-Poisson regression to account for overdispersion. In the regression models, seasonality was accounted for using flexible cubic splines [15] (7 evenly distributed internal knots in weekly data, 5 in monthly data, and boundary knots at the first and last week/month of the year), separate dummy -variables were included to account for clearly observed overall inactivity annually during July and in the last week of every year, and possible secular trend by using an integer vector from 1 to the number of weeks/months included in the data [16].

The hypothesized immediate step-wise change of the COVID-19 lockdown period was modelled by including a dummy variable from 3/16/2020 (or 3/2020 in monthly data) to the end of the year. A separate ascending integer vectorer was included, starting from 5/18/2020 (or 5/2020 in monthly data), to model the hypothesized delayed slope-change after Spring 2020 in the variables of interest [15]. The validity of the regression models was inspected using autocorrelation functions, partial autocorrelation functions and residual plots. Two-sided significance tests and confidence intervals were calculated for the parameters of interest.

We also made counterfactual predictions based on our estimated models to demonstrate the (estimated) continuation of time series without the effects of COVID-19. All the statistical analyses were conducted using R version 4.0.3 [17].

## Results

**Fig. 1:**
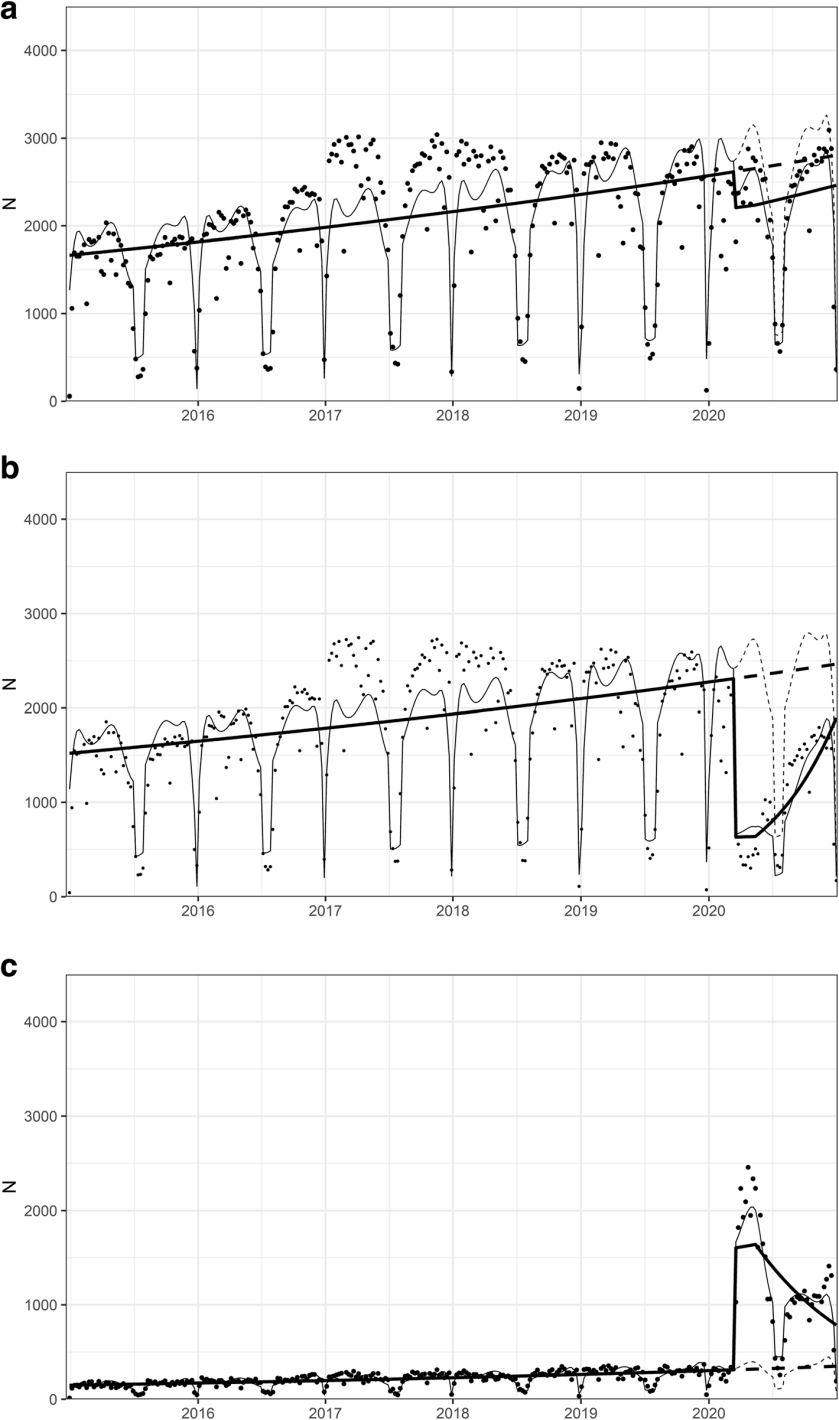
Outpatient visits 2015–2020 (weekly data). **a**) The total number of visits. **b**) The number of in-person visits. **c**) The number of remote visits. The dots denote weekly data points, the fine line denotes the fitted line and the fine dashed line denotes the predicted counterfactual line based on the model (based on data from years 2015–2019). The thick line denotes the trend over time when controlling for seasonality, and the thick dotted line denotes the counterfactual prediction when controlling for seasonality, based on previous 5 years (2015–2019). X-axis shows the time from Jan, 1, 2015 to Dec, 31, 2020, ticks denote the start of each year. Y-axis shows the number of visits

The total number of visits showed a mild decrease in Spring 2020 (step change of − 16, 95% CI -24- -6.4%, *p* < 0.002), whereas later change in slope was not significant, compared with the predicted counterfactual outcome (Fig. 1a). In-person visits decreased in Spring 2020 significantly (step change of − 73, 95% CI -77- -68%, *p* < 0.0001), and after the Spring a significant increase in slope was observed (change with weekly increase of 3.2, 95% CI 2.3–4.1%, *p* < 0.0001) (Fig. 1b). Remote visits sharply increased starting in 3/2020 (step change of 412, 95% CI 370–460%, *p* < 0.0001), and after 5/2020, a significant decrease in slope was observed (weekly change of − 2.5, 95% CI -2.9- -2.1%, *p* < 0.0001); thus, after Summer 2020 remote visits were at a lower level than in Spring, but still at a higher level than predicted based on pre-pandemic data (Fig. 1c). The portion of remote visits of all outpatient visits was 47% during year 2020, whereas in previous 5 years (2015–2019) it was 10–12% (Online Resource, Fig. S1a). Remote visits comprised predominantly of phone calls before the pandemic and at the very early stage of the pandemic (3–4/2020), when they increased significantly (step change of 201, 95% CI 179–225%, *p* < 0.0001), whereas video calls peaked a little later in Spring 2020 (step change of 469, 95% CI 319–697%, *p* < 0.0001) (Online Resource, Fig. S1b). Both modes of remote visits decreased after Spring 2020 (weekly change for phone calls − 2.3, 95% CI -2.7 - -1.9%, *p* < 0.0001, and for video calls − 2.4, 95% CI -3.9 - -0.8%, *p* < 0.003), but remained at a much higher level than in previous years (Online Resource, Fig. S1b).

**Fig. 2:**
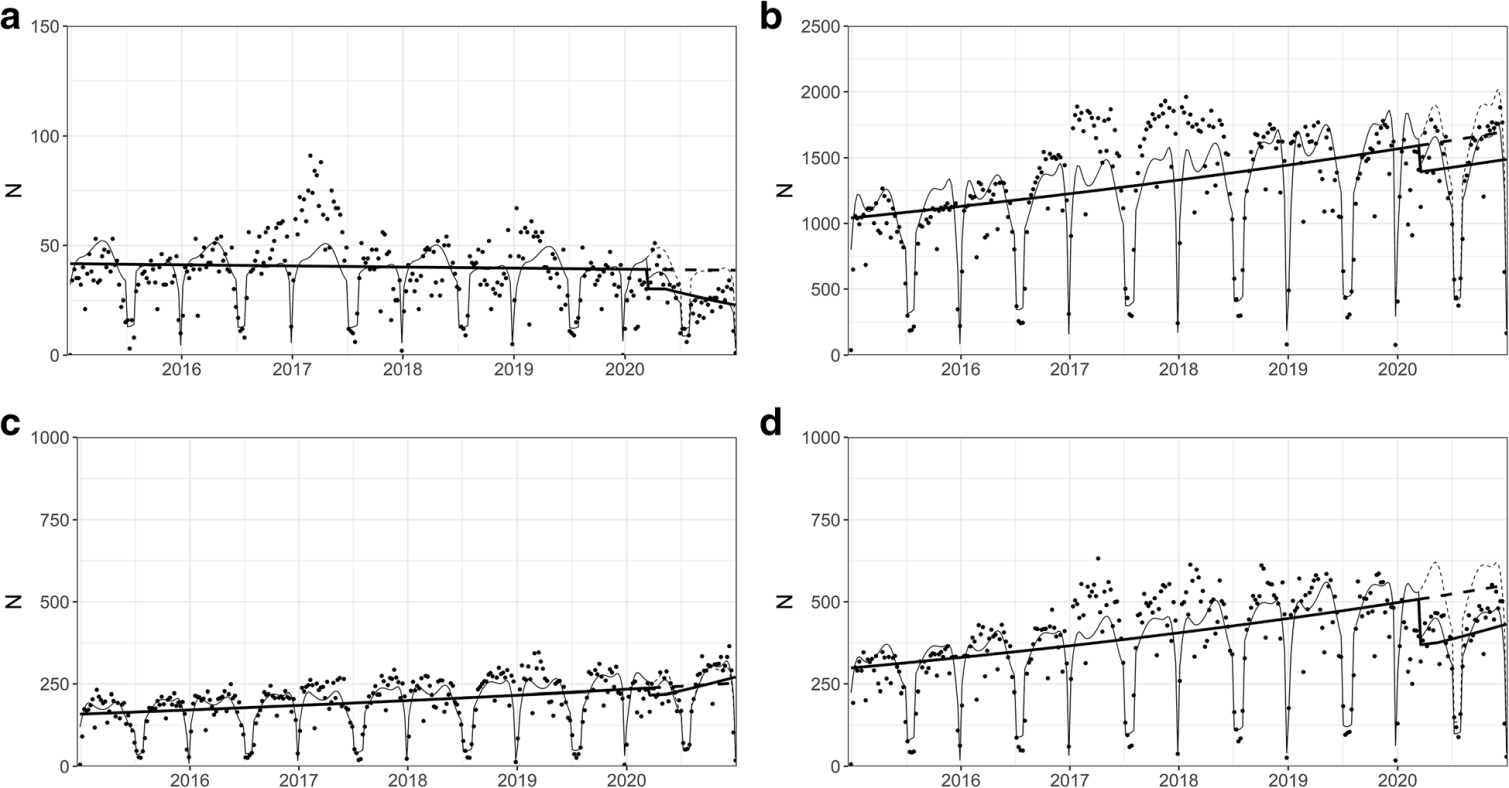
Outpatient visits 2015–2020 in different psychiatric diagnostic groups (weekly data). **a**) psychotic disorders (F20–29). **b**) depressive and anxiety disorders (F32, F33, F40–48, F93.0, F93.1, F93.2, F93.80, F93.9). **c**) ADHD and conduct disorders (F90, F91, F92). **d**) all other psychiatric diagnoses. The dots denote weekly data points, the fine line denotes the fitted line and the fine dashed line denotes the predicted counterfactual line based on the model (based on data from years 2015–2019). The thick line denotes the trend over time when controlling for seasonality, and the thick dotted line denotes the counterfactual prediction when controlling for seasonality, based on previous 5 years (2015–2019). X-axis shows the time from Jan, 1, 2015 to Dec, 31, 2020, ticks denote the start of each year. Y-axis shows the number of visits. NB. The scale of Y axes varies between figures

When we stratified the data according to psychiatric diagnoses coded for the visits, the results mainly conformed to the observations in the aggregate data (Fig. 2a-d). However, in the group of psychotic disorders, the decrease in in-person visits of Spring 2020 was similar, but it was not followed by any significant change in slope unlike in other diagnostic groups (Online Resource, Fig. S2a-d). Changes in remote visits were similar in all diagnostic groups and consequently similar to the changes observed in the aggregate data: rapid increase in Spring 2020 and slow gradual decrease afterwards (Online Resource, Fig. S2d-f).

The number of subjects in adolescent outpatient care on a monthly basis showed a slight but significant decrease in Spring 2020 (step change of − 10, 95% CI -17- -2.9%, *p* < 0.01), and no significant change in slope during the rest of the year 2020 (Online Resource, Fig. S3).

**Fig. 3:**
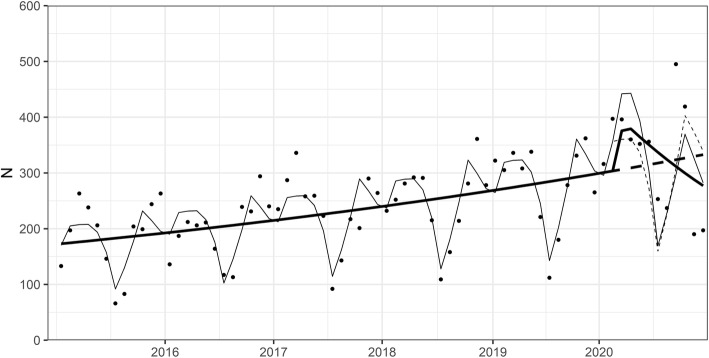
Referrals to HUH adolescent psychiatry outpatient clinic from other health care providers 2015–2020 (monthly data). The dots denote monthly data points, the fine line denotes the fitted line and the fine dashed line denotes the predicted counterfactual line based on the model (based on data from years 2015–2019). The thick line denotes the trend over time when controlling for seasonality, and the thick dotted line denotes the counterfactual prediction when controlling for seasonality, based on previous 5 years (2015–2019). X-axis shows the time from Jan, 1, 2015 to Dec, 31, 2020, ticks denote the start of each year. Y-axis shows the number of referrals

Referrals to HUH adolescent psychiatry outpatient clinic from other health care providers (mostly from primary health care) did not significant change in Spring 2020 compared with previous 5 years, but decreased towards the end of 2020 (weekly change of − 4.7, 95% CI -8.8- -0.5%, *p* = 0.03) (Fig. 3).

## Discussion

Our main finding was that the number of visits at HUH adolescent psychiatry outpatient care at the early stage of the COVID-19 pandemic only slightly decreased compared with counterfactual prediction based on previous 5 years, as rapid increase in remote visits compensated for the steep drop in in-person visits. After Spring 2020, in-person visits began to gradually increase, but the proportion of remote visits of all visits remained at a higher level than before the pandemic. In-person visits of adolescents with psychosis diagnosis did not, however, show any positive gradual change unlike those observed in the aggregate data and other diagnostic groups.

Our result that the total number of visits slightly decreased at the early stage of the pandemic supported our hypothesis, which was based on the assumption that physical distancing measures would interfere with accessing care and on studies reporting a drop in visits during Spring 2020 in primary mental health care [10] as well as in psychiatric emergency visits in adults [11] and in underaged subjects [12]. The change was, however, much smaller in magnitude than the ones reported in the afore mentioned studies, which is explained by the swift transition to offering care remotely. Similar transition in response to the pandemic was reported in secondary outpatient mental health care in the UK especially among underaged patients [14]. The rapid transition from in-person to remote psychiatric outpatient care during the pandemic has been predominantly positively received according to a qualitative study that interviewed psychiatrists [18].

We did not observe any significant increase in visits nor in referrals to adolescent psychiatric outpatient care later during the pandemic, despite many studies reporting increased mental distress during the pandemic in both adult [3, 4] and underaged populations [6–9] and our consequent hypothesis that demand for adolescent psychiatric care would increase during the second half of year 2020. The current study spans up until the end of the year 2020. One possible explanation is that adolescents and their families do not seek help for mental health issues, or primary health care is not able to properly respond to their needs, until after the acute phase of the pandemic has subsided. All in all, any change in psychiatric morbidity in the general adolescent population often affects the demand for secondary and tertiary care in adolescent psychiatry, which is our study setting, with a lag, and thus the potential effect of the pandemic may not yet appear in our study period. Another perspective is that some longitudinal studies have observed mental health to rebound after the restrictions on everyday life ease both among adults [5] and adolescents [7, 8]. Some parents have even seen their child’s mental health improve during the pandemic [9], and adults surveyed during a lockdown expressed the situation to entail diverse positive aspects for mental health [19].

Our observation that outpatient in-person visits gradually returned near to the expected levels after lockdown is in line with data on secondary outpatient mental health care in the UK [14]. From June, 2020, onwards HUH recommended remote visits in outpatient care, but patients were allowed to opt for in-person visits based on subjective preference. Our result would thus suggest that in-person visits were preferred over remote visits in our study population, contrary to findings of an Australian study where adolescents attending mental health services rated high satisfaction with telehealth and expressed interest in continuing its use after the pandemic [20]. Research on adult populations shows that telephone and video-delivered synchronous interventions in mental health care are as effective as in-person care [21], whereas research on adolescents lags behind [22]. Both quantitative and qualitative research in adolescent populations on remote mental health care addressing issues like the effectiveness of care, participants’ satisfaction and best practices would be most welcome in order to improve the flexibility of services without sacrificing quality.

The result in our study that raises concern and opposes to our hypothesis is that in-person visits of adolescents with a diagnosed psychotic disorder remained at a low level during the pandemic after Spring 2020 contrary to other diagnostic groups. Remote visits in this patient group increased in Spring 2020, but like among most patients, they later gradually decreased. In clinical practice, adolescents with psychosis and their families would need, in the light of our results, more support than other patients in accessing care during a pandemic. Research on adults shows that remote care is feasible and effective in assessing and treating patients with psychosis [23], but research on underaged patients with psychosis is lacking [22]. A further research topic could be how adolescents with psychosis, and their families, experience return to in-person visits after a lockdown and what kind of support they might need.

Strength of the study is the relatively long time period of 5 years we compared the pandemic period to, and also the relatively long time span, in comparison to other studies published so far, of the pandemic from the onset until the end of year 2020. A limitation is that the study is based on one organization only, but on the other hand HUH adolescent psychiatry is the largest secondary mental health care unit for adolescents in Finland. The data inevitably reflect some organizational issues such as variation in personnel resources and rare events such as the transition to a new clinical software in 2019–2020. We sought to compensate these limitations with the rather long comparison period of 5 years. The number of visits by adolescents with a psychotic disorder was low and the results are not as robust as in other diagnostic groups with more patients and visits. Our study was set in secondary and tertiary adolescent psychiatric care, and thus not directly comparable to studies on primary or mixed level mental health care. In our statistical analyses, we sought to model the dynamics of the pandemic period by including both step and slope change, but we recognise that interpreting statistical significance of such analyses may be problematic [24]. Finally, a rapid switch to remote care such as the one we observed requires high level of internet access and acceptability of online services as well as confidence in health care service providers, which are features of the Finnish society. Our results should be compared with caution to observations from societies that differ in these aspects.

Our results demonstrate that mental health services need to be flexible and responsive in how care is delivered in the event of a disruptive and evolving phenomenon such as the COVID-19 pandemic. Special attention should be given to most vulnerable and severely ill patients such as those with a psychotic disorder. Longitudinal studies on mental health outcomes and care reaching over to the post-pandemic period will show whether access to care and effectiveness of (mainly remote) treatments have been sufficient during the pandemic. Given that adolescence is such a critical developmental stage where social relationships are key, both clinical and research efforts need to specifically address this age group during the pandemic and its aftermath.

## Supplementary Information

**Additional file 1:** **Fig. S1.** Outpatient in person and remote visits on annual level, and division of remote visits to phone calls and video calls. **a)** Annual number of outpatient visits and the proportion of in-person (blank) and remote (shaded) visits 2015–2020. X-axis shows the year and Y axis the number of visits. **b)** Remote visits (thick line) comprised of phone calls (dashed line) and online video calls (fine line). X-axis shows the time from Jan, 1, 2015 to Dec, 31, 2020, ticks denote the start of each year. Y-axis shows the number of visits.

**Additional file 2:** **Fig. S2.** Outpatient in person and remote visits 2015–2020 in different psychiatric diagnostic groups. **a)** In person visits in the group of psychotic disorders (F20–29). **b)** In person visits in the group of depressive and anxiety disorders (F32, F33, F40–48, F93.0, F93.1, F93.2, F93.80, F93.9). **c)** In person visits in the group of ADHD and conduct disorders (F90, F91, F92. **d)** In person visits in the group of all other diagnoses. **e)** remote visits in the group of psychotic disorders (F20–29). **f)** remote visits in the group of depressive and anxiety disorders (F32, F33, F40–48, F93.0, F93.1, F93.2, F93.80, F93.9). **g)** remote visits in the group of ADHD and conduct disorders (F90, F91, F92). **h)** remote visits in the group of all other diagnoses. The dots denote weekly data points, the fine line denotes the fitted line and the fine dashed line denotes the predicted counterfactual line based on the model (based on data from years 2015–2019). The thick line denotes the trend over time when controlling for seasonality, and the thick dotted line denotes the counterfactual prediction when controlling for seasonality, based on previous 5 years (2015–2019). X-axis shows the time from Jan, 1, 2015 to Dec, 31, 2020, ticks denote the start of each year. Y-axis shows the number of visits. NB. The scale of Y axes varies between figures.

**Additional file 3:** **Fig. S3.** The number of subjects in adolescent outpatient care 2015–2020 (monthly data). The dots denote monthly data points, the fine line denotes the fitted line and the fine dashed line denotes the predicted counterfactual line based on the model (based on data from years 2015–2019). The thick line denotes the trend over time when controlling for seasonality, and the thick dotted line denotes the counterfactual prediction when controlling for seasonality, based on previous 5 years (2015–2019). X-axis shows the time from Jan, 1, 2015 to Dec, 31, 2020, ticks denote the start of each year. Y-axis shows the number of subjects.

## Data Availability

The datasets used and analysed during the current study are available from the corresponding author on reasonable request.

## Abbreviations

ADHD: Attention-Deficit/Hyperactivity Disorder
COVID-19: Coronavirus disease of 2019
HUH: Helsinki University Hospital
ICD-10: International Classification of Diseases 10th Revision
WHO: World Health Organization
