# Supplementary figures and images for: Adolescent psychiatric outpatient care rapidly switched to remote visits during the COVID-19 pandemic
Source: BMC Psychiatry. 2021 Nov 20;21:586. doi: 10.1186/s12888-021-03580-w (PMC8605888; doi:10.1186/s12888-021-03580-w)

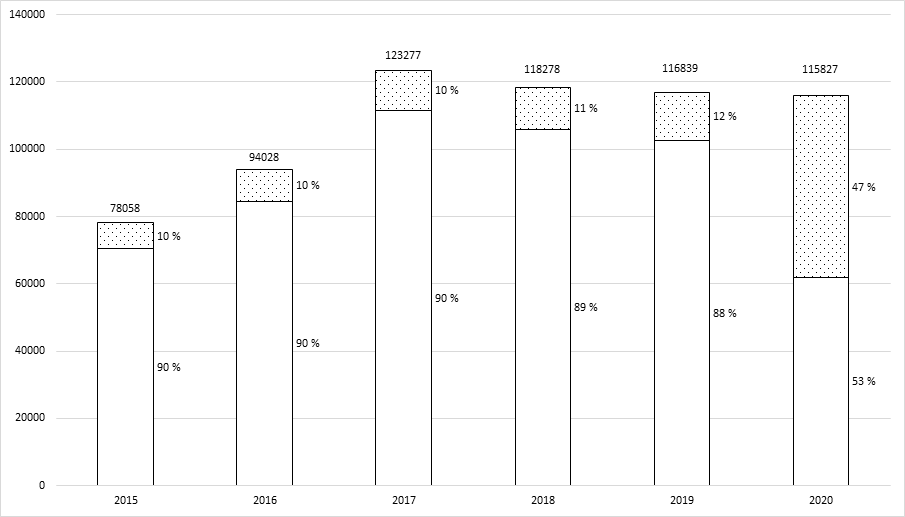

Supplement: Supplementary file 1 — Additional file 1: Fig. S1. Outpatient in person and remote visits on annual level, and division of remote visits to phone calls and video calls. a) Annual number of outpatient visits and the proportion of in-person (blank) and remote (shaded) visits 2015–2020. X-axis shows the year and Y axis the number of visits. b) Remote visits (thick line) comprised of phone calls (dashed line) and online video calls (fine line). X-axis shows the time from Jan, 1, 2015 to Dec, 31, 2020, ticks denote the start of each year. Y-axis shows the number of visits. [file 12888_2021_3580_MOESM1_ESM.zip › figS1aR2.png]
